# Management of gall bladder perforation evaluation on Ultrasonography: report of six rare cases with review of literature

**Published:** 2011-11-24

**Authors:** Singal Rikki, Mittal Amit, Gupta Samita, Singh Bir, Jain Parul

**Affiliations:** * Department of Surgery- Maharishi Markandeshwer Institute of Medical Sciences and Research, Mullana, (Distt-Ambala), Haryana, India; ** Department of Radiodiagnosis and Imaging- Maharishi Markandeshwer Institute of Medical Sciences and Research, Mullana (Distt -Ambala) Pin Code 133201, Haryana, India; *** Department of medicine - Maharishi Markandeshwer Institute of Medical Sciences And Research, Mullana, (Distt-Ambala), Haryana, India

**Keywords:** ultrasonography, acute cholecystitis, peritonitis

## Abstract

Background: Perforation of the gall bladder with cholecystohepatic communication is a rare cause of liver abscess. We are reporting here six rare cases of gall bladder perforation with variable clinical presentations.

Materials and Methods: Most patients presented with right hypochondrium pain and fever but two patients presented with only pain in the abdomen. Ultrasonography (USG) and Computed Tomography (CT) were used for diagnosis. The patients were also successfully treated.

Results: There was a gall bladder perforation with cholecystohepatic communication, leading to liver abscess formation in most cases on USG and CT. The final diagnosis was confirmed on surgery.

Conclusion: The perforation of the gall bladder which leads to liver abscess is a rare complication of acute, chronic or empyema gall bladder. USG and CT scans are the most important diagnostic tool in diagnosing this rare complication. In the set up, where advanced options are not available, the only treatment of choice is the conservative one or surgery, according to the status of the patients.

## Introduction

The perforation of the gall bladder with cholecystohepatic communication is a rare cause of liver abscess [**1,2**]. In 1934, Neimeir proposed a classification of the acute perforation of the gall bladder based on his findings and therapeutic approaches (**Table 1**) [**3,4**].

**Table 1:** In 1934 Niezmer classified Perforations of the gall–bladder into three groups

Type 1 (acute) is associated with generalized biliary peritonitis,
Type 2 (subacute) consists of the localization of fluid at the site of perforation, pericholecystic abscess and localized peritonitis, while
Type 3 (chronic) comprises the formation of internal or external fistulae

He concluded that this rare condition required early recognition and treatment to decrease the mortality rate. There are no classical symptoms and signs associated with gall bladder perforations (GBP). Intrahepatic perforation of the gall bladder, with liver abscess and cholecystohepatic communication, is even rarer. Only a few adult cases have been reported in the English literature [**5,6**]. Ultrasonography (USG) is the initial imaging modality of choice for the evaluation of the suspected acute gall bladder disorders, and it is often sufficient for a correct diagnosis. Contrast enhanced computed tomography (CECT) also plays a vital role in the evaluation of acute gall bladder pathology. CT is particularly useful in situations where ultrasound findings are equivocal. CT is also extremely valuable in the assessment of suspected complications of acute cholecystitis, particularly emphysematous cholecystitis, hemorrhagic cholecystitis, and gall bladder perforation, diagnoses which are often very difficult to establish at sonography [**7**].

## Materials and methods

**Case Report 1:** A 40–year–old female presented with a case of acute abdomen. She had acute pain in the abdomen and had been vomiting for two days. Fever was also present. Pain was more in the right hypochondrium region. The pulse rate of the patient was of 120/min and blood pressure–120/70 mm of Hg. On abdominal examination, generalized tenderness was present mainly at the right upper quadrant region. Rigidity was present in the right upper abdomen but no mass was felt. On laboratory findings, white cell counts were increased. Rests of blood tests and liver function tests were within normal limits. Chest X–ray revealed no gas under diaphragm.

**Fig. 1:**
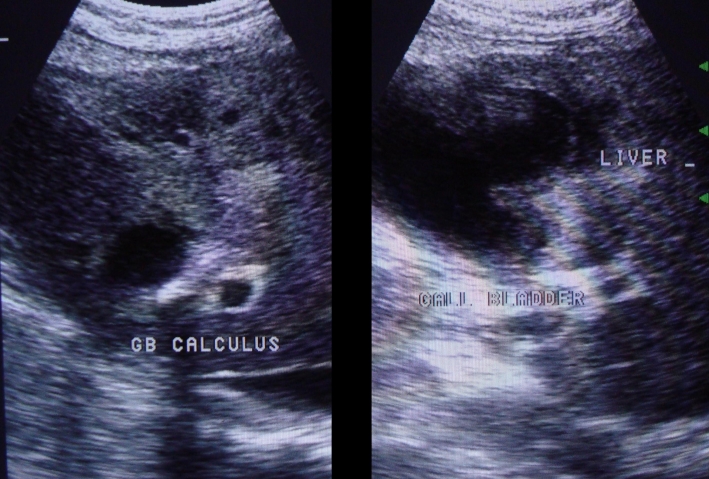
Ultrasonography is showing abscess formation in the liver due to gall bladder perforation

The USG of the abdomen revealed distended gall bladder with thickened wall (5mm) and thick internal echoes with a large 1.2 cm calculus in the neck of gall bladder suggesting cholelithiasis with empyema of gall bladder. There was a large defect of 2 cm in the left anterior wall of gall bladder with the formation of large abscess in adjacent liver. Based on these findings, a diagnosis of perforation of gall bladder with formation of liver abscess was made (**Fig. 1**).

**Fig. 2:**
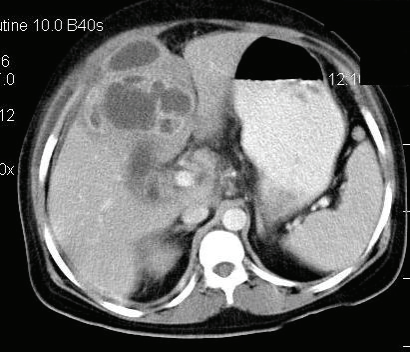
Contrast enhanced computed tomography of the abdomen is showing gall bladder empyema with perforation and formation of liver abscess.

CECT scan of abdomen showed gall bladder perforation with formation of liver abscess (**Fig. 2**).

The patient was operated and empyema gall bladder that communicated with the liver was detected. There was a single large stone (of approximately 1.5 cm) in the cystic duct and common bile duct was dilated. Adhesions were present to surrounding structures. Bile was found in peritoneal cavity. Cholecystectomy was done and abdominal cavity was thoroughly washed with normal saline. The patient had no specific problems in the follow up period of 6 months after the operation.

**Fig. 3:**
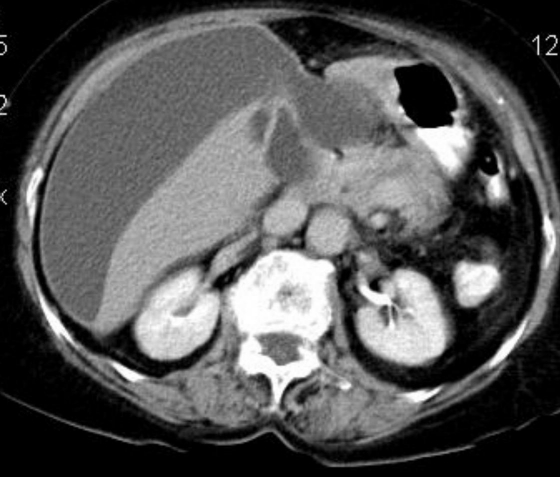
Contrast enhanced computed tomography scan of the abdomen revealed abdomen revealed gall bladder perforation with perihepatic collection.

**Case report 2:** A 65–year–old female reported to emergency with painful abdomen, fever and loss of motion. Pain had been dominant in the right hypochondrium region for one month and an exacerbation of pain was evident for the last 15 days. The patient was taking medicine from a private practitioner. Fever and vomiting were present for the last 7 days. Fever was present in the evening and she took medicines to lower it. At examination, the blood pressure was of 100/60 mm of Hg and the pulse rate was of 96/min. There was no jaundice or anemia. On local examination, generalized tenderness was found in the whole abdomen. Rigidity was present. Bowel sounds were also present. The provisional diagnosis made was cholecystitis. While on investigation, routine blood tests were within normal limits including the liver function tests. There was no free gas under the diaphragm and an abdominal X–ray was reported as normal. Ultrasonography revealed a perforation in the gall bladder with perihepatic fluid collection. There was no calculus in the gall bladder and thickness of gall bladder wall was normal. CECT abdomen revealed a gall bladder perforation with perihepatic collection, however, there was no evidence of inflammation or collection (**Fig. 3**).

Intrahepatic hepatic biliary radicals (IHBR) and common bile duct (CBD) were normal. Based on USG and CT the diagnosis made was of spontaneous perforation of acalculous gall bladder. Laparotomy was done. Operative findings revealed a quantity of approximately 200 ml of bile in the right hypochondrium perihepatic space with a perforation from the gall bladder fundus. There was no evidence of inflammation or calculus. So, the radiological findings correlated with the operative findings. The rest of the abdominal parts were normal.

**Case report 3:** A 33–year–old female presented with vomiting, abdominal pain, absolute constipation and hyperpyrexia for five days. Abdominal examination revealed rigidity, guarding, rebound tenderness and absence of bowel sounds. An erect skiagram showed dilated loops of small bowel. Ultrasound revealed aperistaltic bowel loops with two large calculi in the ileum (3 and 1 cm) and proximal small gut obstruction. There was also evidence of air in the gall bladder fossa with contracted gall bladder (**Fig. 4,Fig. 5**).

Fig. 4 and Fig. 5 Ultrasound revealed aperistaltic bowel loops with two large calculi of size 3 and 1 cm and proximal small gut obstruction in the ileum

**Figure:**
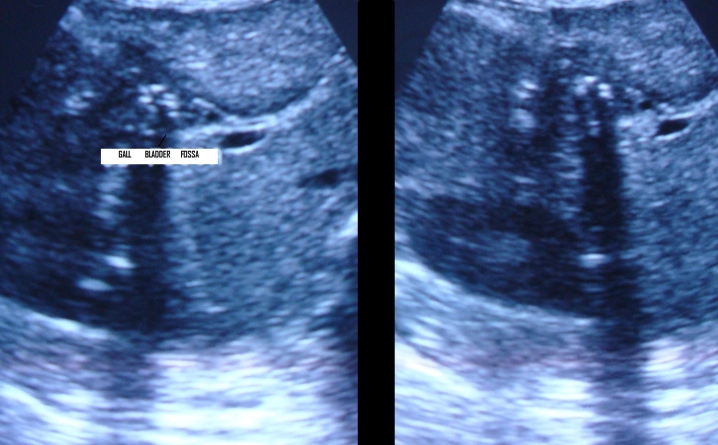

**Figure:**
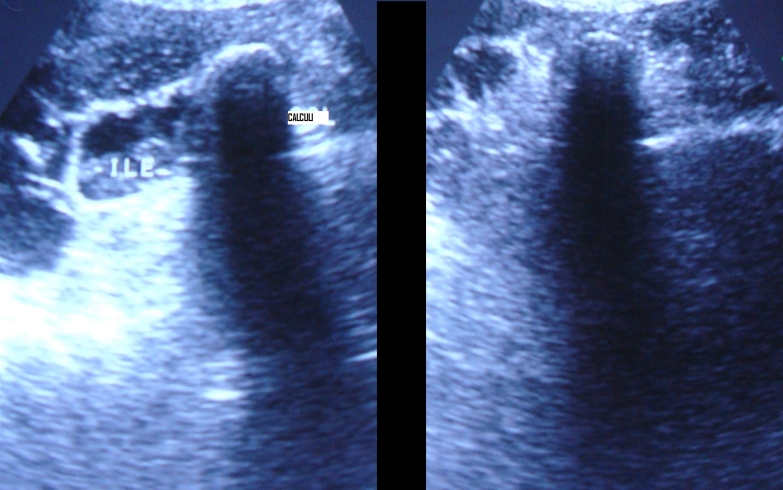

A diagnosis of gall stone ileus with cholecysto–enteric fistula was made. On laparotomy, two large calculi were seen in the distal ileum causing obstruction of the proximal gut. The calculi were removed from the ileum only by enterolithotomy. The patient recovered well postoperatively. On follow up after 6 months, the patient was very well.

**Case report 4:** A 53–year–old female presented to the emergency department with painful abdomen and vomiting. Pain was present for 1 month and it was non-radiating. Pain was increased after food intake. Vomiting was present for the last week. There were no other complaints. The patient was passing motion.Vital signs were stable. On the examination of the abdomen, tenderness was present only in the right hypochondrium region, the rest of the examination being normal. Bowel sounds were sluggish.

**Fig. 6:**
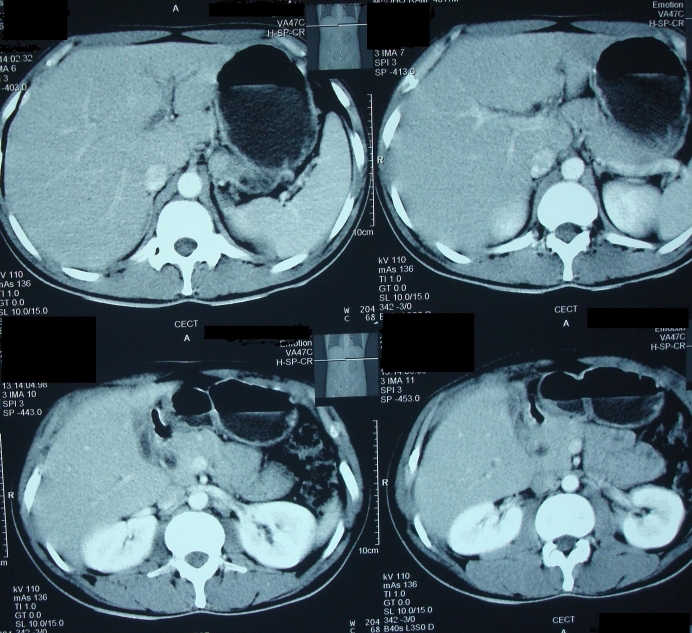
Contrast enhanced computed tomography (CECT) of the abdomen revealed gall bladder perforation with minimal peri-cholecystic collection and dilated hepatic ducts

USG of the abdomen revealed a rent in the medial wall of the gall bladder with very minimal pericholecystic collection. IHBR and CBD were prominent with a small calculus of 5 mm in proximal CBD. CECT of the abdomen revealed gall bladder perforation with minimal pericholecystic collection and dilated hepatic ducts (**Fig. 6**).

The patient was kept under conservative treatment. She was not given any food orally. She was put on third generation antibiotic with amikacin (1 gm twice/day) and metrogyl with intravenous fluids. She started oral liquids on the 5th day of the conservative treatment. While repeating the USG done after 5 days, the CBD calculus was not found and the CBD and IHBR became normal.

**Fig. 7:**
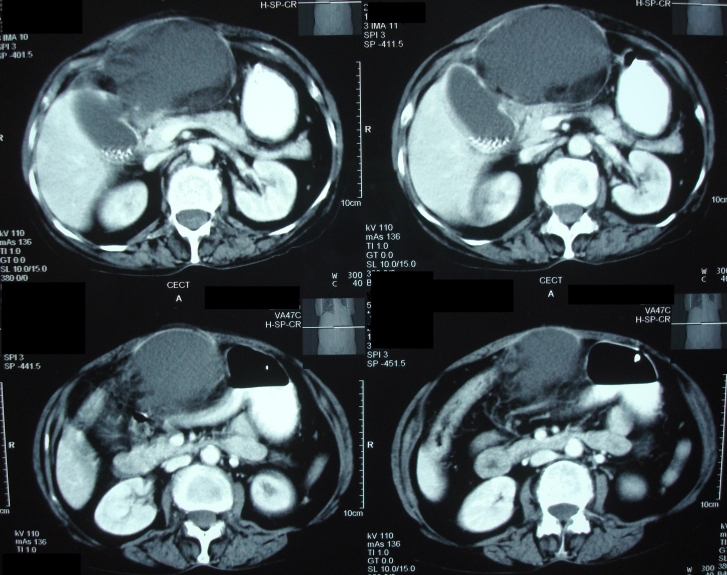
CECT abdomen revealed multiple calculi in distended gall bladder, with 6 mm perforation in the medial wall of gall bladder and large abscess formation in the anterior abdominal wall

**Case report 5:** A 67–year–old female reported to the emergency with painful abdomen that had been persisting for 5 days. Pain was severe in nature, but non–radiating. There was history of vomiting for 4 days with loose stools and high degree fever for the last two days. No other complaints were present. Blood pressure was of 90/56 mm of Hg. Tachycardia and tachypnoae were present. Temperature was raised. Routine blood investigations were within normal limits including liver function tests, except for the raised total leucocyte counts (36,900/cu mm). X–ray of the chest and abdomen were normal. USG of the abdomen revealed a rent of 6–8 mm in the left lateral wall of the gall bladder, extending to subcutaneous region with large abscess collection seen in the anterior abdominal wall, in the epigastrium. CECT abdomen revealed multiple calculi in distended gall bladder, with 6 mm perforation in the medial wall of gall bladder and large abscess formation in the anterior abdominal wall, with severe surrounding inflammation (**Fig. 7**).

**Fig. 8:**
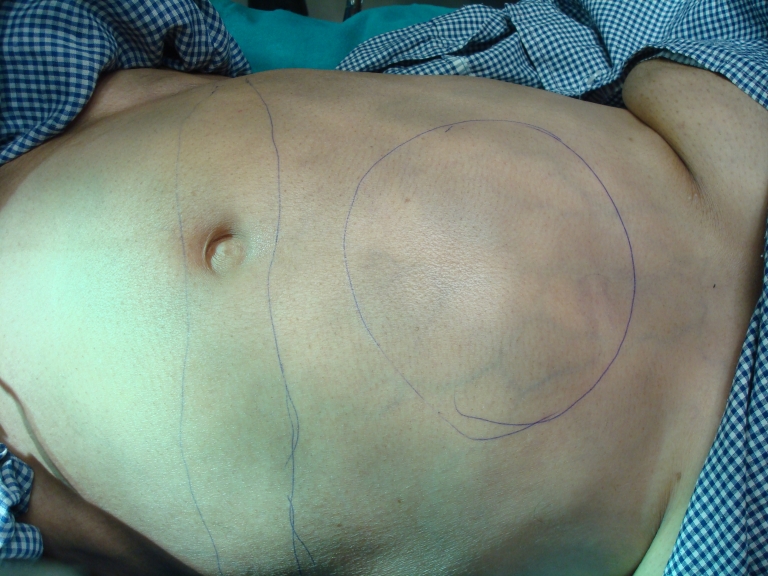
A lump in the right hypochondrium and subcostal region near to epigastrium

Local examination of the abdomen revealed a lump in the right hypochondrium and subcoastel region near to epigastrium (**Fig. 8**).

The lump was soft in consistency and tender in nature. Gaurding was present in the right side of the abdomen. Bowel sounds were absent. Bilateral coarse crepitations were present on auscultation of the chest.

**Fig. 9:**
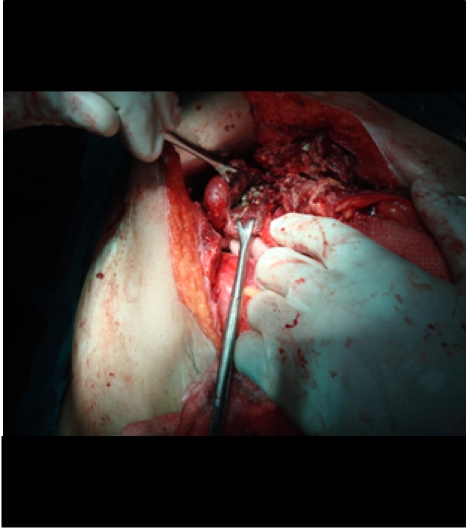
Operative findings revealed pus in the right subcostal region with erosion of the sheath

After a proper resuscitation, the patient was taken for an emergency laparotomy. Operative findings revealed pus in the right subcoastel region with erosion of the sheath (**Fig. 9**).

There were dense adhesions to the gall bladder. Omentum was necrosed. A perforation was seen in the body of the gall bladder with spillage of the stones in the subcostal space (**Fig. 10 a, b**).

**Figure:**
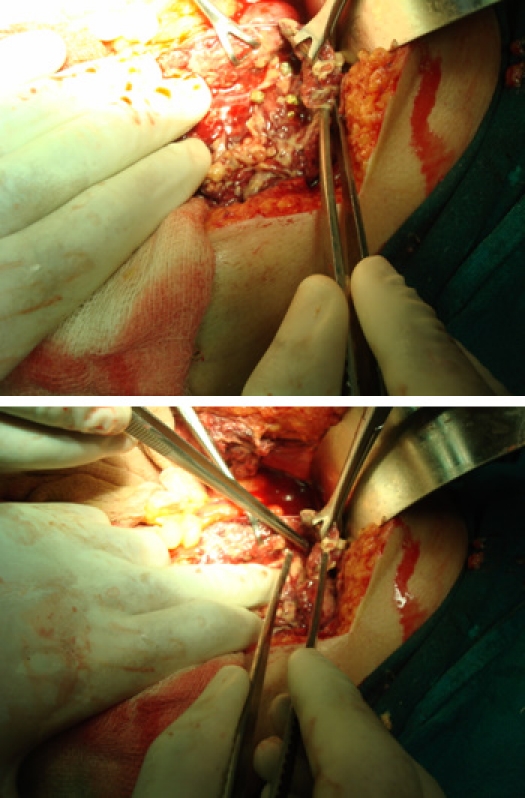
Fig. 10 a and b A perforation was seen in the body of the gall bladder with spillage of the stones in the subcostal space

After opening the gall bladder, multiple gall stones were found. Cholecystectomy was done by washing. A drain was kept in the subcostal area. Post-operatively, the patient was kept on SIMV mode for two days because of the chest spasm. The patient started taking on the 7th post-operative day and oxygen saturation was maintained to 99%. She was discharged on the 10th day from surgery. In the follow up, patient was asymptomatic and was doing well.

**Case report 6:** A 45–year–old female was admitted with complaints of severe pain in the abdomen and fever for 4 days. The pain was located more in the epigastrium and right hypochondrium region without any vomiting. Fever was present for 4 days more in evening without any rigor and chills. The patient was admitted in a private hospital before one month for abdominal pain. She was diagnosed with acute cholecystitis, for which, conservative treatment was given.

**Fig. 11:**
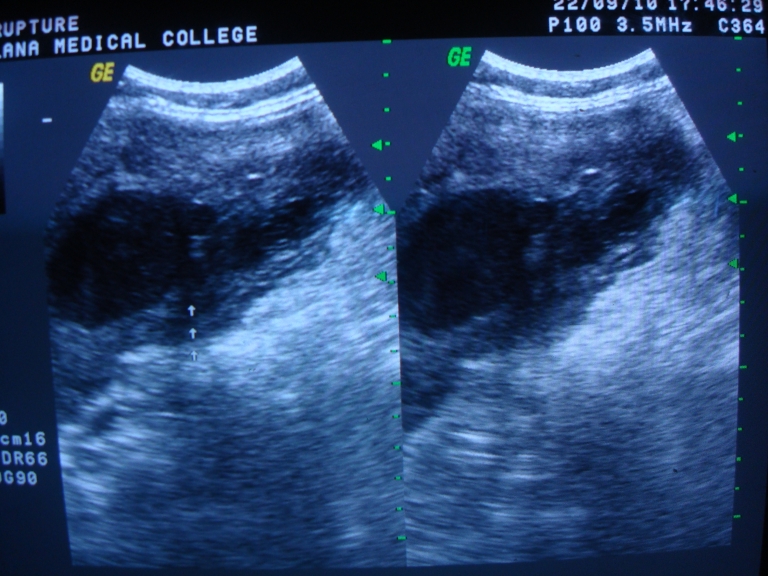
Ultrasonography of abdomen showed a perforation in fundus without any collection

Tenderness was present in the right hypochondrium region on examination without any rigidity or guarding. Bowel sounds were present. Routine blood tests were high, including liver function tests. Serum lipase was high. There was a perforation in fundus without any collection on USG of abdomen (**Fig. 11**).

Chest X–ray was normal. The patient was treated successfully with conservative treatment by third generation antibiotics and metrogyl.

## Discussion

Asymptomatic cholelithiasis is a frequent condition which affects up to 10% of the adult population in wealthy nations. Acute cholecystitis develops in up to 2% of patients affected by asymptomatic cholelithiasis. GBF occurs in 2 to 11% of acute cholecystitis cases [**8**]. Perforation of the gall bladder is an infrequent and serious complication of cholecystitis and a delay in the correct diagnosis. Also, the mortality is high even after different interventions. GBF represents a special diagnostic and surgical challenge [**8,9**]. The gall bladder has a dual blood supply. Hence, though acute cholecystitis is common, avascular necrosis and gangrene with perforation is relatively uncommon. In the presence of generalized vascular insufficiency, localized tissue ischemia leads to cellular dysfunction. The gall bladder fundus is most common site for perforation. The primary origin of acute cholecystitis is the persistent occlusion of the cystic duct by an impacted stone that causes increased gall bladder wall tension, epithelial injury, release of phospholipases, degradation of adjacent cell membranes, and intense inflammatory reaction [**10**]. The incidence of this complication has shown a decline over time since its first description by Duncan in 1884 [**11**]. Rates of GBF are decreased due to an increase in the number of cholecystectomies performed as compared with the past ones. A recent case series found the rate of perforation to be of 0.8% [**12**]. Overall, perforation of the gall bladder from acute cholecystitis occurs in approximately 3–10% of patients. Usually, it manifests as acute free perforation, subacute perforation with pericholecystic abscess, or chronic perforation with cholecystoenteric fistula and, occasionally, gallstone ileus.

In 1934, Niemeier classified GBF into three types: type 1, chronic perforation with the presence of a fistulous communication between the gall bladder and some other viscus; type 2, subacute perforation where the perforated gall bladder is surrounded by an abscess walled off by adhesions from the general peritoneal cavity; and type 3, acute perforation of the gall bladder into the free peritoneal cavity without protective adhesions (**Table 1**) [**13**].

Somasekhar RM et al [**5**] reported 31 patients with GBF. Nine patients (29%) had type I perforation, presented with complaints of generalized abdominal pain. Seven patients had history of pain, which was initially localized to the right hypochondrium before becoming generalized. Three of these were already diagnosed to have gallstone disease and were waiting for elective cholecystectomy. Seven (78%) patients had presented with shock. Features of generalized peritonitis were seen in six (67%) patients. Type II perforation was present in 14 patients (45%) who presented with complaints of right hypochondrial pain, which ranged in duration from 3 days to 15 days. All except for one patient described a history of such complaints even though they may have been milder. Type III perforation was present in eight patients (26%) and presented with acute cholecystitis. However, these patients had symptoms of longer duration and were recurrent. Fever was noted in 15 (48%) patients, all of whom had either type I or type II perforations. An abdominal mass was present in 11 patients (36%) while three (10%) patients have presented with jaundice.

GBF can be due to cholelithiasis, infection, malignancy, trauma, corticosteroid therapy, diabetes mellitus, impaired vascular supply, old age and male sex. Patients may present with right upper quadrant pain, fever, palpable right upper quadrant mass and tenderness may herald an acute onset. However, patients may present with weakness, malaise, anorexia and a palpable right upper quadrant mass in malignancy [**1**]. Elevated liver enzymes, especially alkaline phosphatase levels are commonly observed [**2,3**]. A sudden decrease in pain intensity may occur due to perforation which causes relief of high intracholecystic pressure [**3**].

The treatment modality, which has not been fully established for GBF with cholecystohepatic communication, leads to liver abscesses, which are rare [**1–3**]. USG may show gall bladder wall thickening >3mm, gall bladder distention (largest diameter >3.5–4.0 cm), gallstones, coarse intracholecystic echogenic debris and bile duct dilatation including halo sign, which can help in diagnosis, as diagnosed in our case [**14,15**]. The earliest signs of impending gall bladder perforation detectable on sonography may be distended gall bladder and edema of its walls along with liver abscess, raise the suspicion of intrahepatic perforation [**16**]. Computed tomography can demonstrate either calculi outside the gall bladder or a ruptured segment of the gall bladder wall, which are the direct indicators of perforation according to Pedrosa et al [**17**].The demonstration of an abscess outside the gall bladder, presence of gallstones and thickening of gall bladder wall are the indirect indicators. Similar to our reported case we also diagnosed a hole sign between the gall bladder and the liver abscess, which matched to other reported studies [**16**]. Fischer [**18**] reported 17 patients with hepatic abscess, out of which two were associated with acute cholecystitis. These hepatic abscesses were found in surgery to be the result of direct extension from an acutely inflamed gall bladder.

Zerman [**19**] reported 5 cases of hepatic abscess secondary to acute cholecystitis (4 males and 1 female), aged between 46 and 78 years. They presented with fever, abdominal pain and, one, with jaundice. Liver abscess were diagnosed by USG and CT scan. In acute phase, all the patients were treated with percutaneous drainage and subsequently, four of them had elective cholecystectomy. CT may have an important role in preoperative diagnosis of complicated acute cholecystitis and in the surgical management of patients with this condition, if ultrasound examination fails to establish the diagnosis or cannot be done for technical reasons [**17**]. Similar to our cases, CT and USG were the helpful investigations in the treatment of the perforation. The increase in incidence may be attributed to better diagnostic modalities like ultrasound and computerized tomography, which have since become available and have been used in the diagnostic evaluation of these patients [**20**].

With CT, a pyogenic liver abscess typically appears as a well–defined, round, hypodense mass with a central density that is between 0–45 Hounsfield units. The peripheral wall usually enhances with contrast administration and internal septa may be present. Small microabscesses may coalesce to form a larger abscess. At sonography, a pyogenic abscess usually appears round or ovoid with variable internal echogenicity [**20,21**].

Type III patients, present with features similar to those of chronic cholecystitis are so are difficult to identify preoperatively, unless they have obstructive symptoms. This has been the experience of other authors as well. Gallstone ileus was first described by Bartolin in 1654 [**26,27**] as an uncommon surgical emergency exclusively in the seventh and eighth decade, sparingly occuring in younger patients. The gallstone intermittently obstructs the bowel before impaction, leading to tumbling obstruction [**28**]. In a review of 458 cases, only two cases of perforation were cited [**29**]. The perforation occurs either at the site of impaction of gallstone, or at previous sites of obstruction and is present because of pressure necrosis of jejunal wall [**30**].

In the past, clinical and radiological aids were insufficient to clinch the diagnosis, however advent of CT and MRI has made it easier [**31**]. Though ultrasound could not specifically identify type I perforations, it was helpful in determining the need for surgical intervention, as it could identify the presence of free fluid, the appearance of which, on guided tap, left no doubts about the pathology. The results of ultrasound and computerized tomography in our patients are similar to those quoted in various studies comparing efficacy of ultrasound and CECT in detecting gall bladder perforation [**22–24**].

Roslyn and Busutte [**32**] have reported mortality rate to be between 12 and 16%. They have suggested that spontaneous gall bladder perforation is caused by hypoperfusion of viscera, secondary to systemic disease and also reported that the fundus of the gall bladder is the most common site of perforation in gall bladder. However, other hypotheses include trauma, congenital abnormality, infection, pancreatic secretions, obstructions, calculi and abnormal bile [**33**].

Although one and two stage procedures can be carried out safely in cases of gall stone ileus with optimal survival, enterolithotomy alone is the minimal surgery sufficient in emergency situations [**34**]. It is an adequate procedure for elderly patients, in which subsequent cholecystectomy is not mandatory [**35,36**]. But, our 3rd case of bowel resection anastomosis was done due to gangrenous and perforation of the bowel to avoid further mortality. In the 4th case, the patient was treated conservatively and was operated after 3 months and discharged in satisfactory condition.

Delay in surgical intervention is the major reason for increased morbidity and mortality associated with GBF. Emergency cholecystectomy should be considered in patients with acute cholecystitis at an early stage to prevent this complication.

We presented a rare case of gall bladder perforation which led to formation of hepatic abscess with cholecystohepatic communication. Initially, such cases could be treated conservatively with percutaneous drainage with subsequent cholecystectomy. Early diagnosis of gall bladder perforation and immediate surgical intervention are of prime importance in decreasing morbidity and mortality associated with this condition. In such cases, the intrahepatic nature of the gall bladder can lead to difficulty in laparoscopic cholecystectomy, with a high rate of conversion to open cholecystectomy.
